# *“Encyclopaedia Cloacae”*—Mapping Wastewaters from Pathogen A to Z

**DOI:** 10.3390/microorganisms13081900

**Published:** 2025-08-15

**Authors:** Aurora Hirvonen, Sara Comero, Simona Tavazzi, Giulio Mariani, Caterina Cacciatori, Roberta Maffettone, Francesco Pierannunzi, Giulia Panzarella, Luis Bausa-Lopez, Sorin Sion, Tanja Casado Poblador, Natalia Głowacka, Davey L. Jones, Mauro Petrillo, Antonio Marchini, Maddalena Querci, Bernd Manfred Gawlik, on behalf of the Encyclopaedia Cloacae Collaborators

**Affiliations:** 1 Joint Research Centre, European Commission, 21027 Ispra, Italy; aurora.hirvonen@ec.europa.eu (A.H.); sara.comero@ec.europa.eu (S.C.); 2 European Dynamics S.A., 54627 Thessaloniki, Greece; 3 SEIDOR S.A., 08019 Barcelona, Spain; 4 Techfusion SRL, 030508 Bucarest, Romania; 5 SEIDOR S.A., 21027 Ispra, Italy; 6 School of Environmental and Natural Sciences, Bangor University, Bangor LL57 2DG, UK; d.jones@bangor.ac.uk

**Keywords:** wastewater-based epidemiology, public health, One Health, *Encyclopaedia Cloacae*

## Abstract

The *Encyclopaedia Cloacae* is a novel and centralised digital platform designed to support and advance wastewater-based epidemiology (WBE) by cataloguing pathogens detectable in wastewater and their relevance to public health surveillance. The platform is hosted on the EU Wastewater Observatory for Public Health (EU4S) website, where it is populated with peer-reviewed research through a structured workflow under harmonised criteria which address the presence of pathogens in human excreta, detectability in wastewater, and integration into public health systems. This tri-criteria approach ensures that the database is both scientifically robust and operationally actionable. Complemented by the *Visualising the Invisible* dashboard, the platform offers geospatial insights into global WBE research activity. By consolidating peer-reviewed evidence on pathogen detectability in wastewater and human excreta, the *Encyclopaedia Cloacae* enables early detection of infectious diseases, whether already known or newly emerging. The continuously updated repository and geospatial dashboards help to identify surveillance gaps and research hotspots, to support timely public health responses, enhance pandemic preparedness, and strengthen global health security. In addition, it supports One Health strategies, connecting the health of humans, animals, and the shared environment. This article outlines the platform’s architecture, data curation methodology, and future directions, including automation and expansion to encompass broader health determinants such as antimicrobial resistance and chemical hazards.

## 1. Introduction

The global health landscape is experiencing a transformative paradigm shift in disease surveillance driven by the need for timely, scalable, and non-invasive monitoring tools. Wastewater-based epidemiology (WBE) has emerged as a powerful approach for assessing population-level health trends by detecting biological markers in community wastewater [1,2]. Its utility in pandemic preparedness has been increasingly recognised, especially since the COVID-19 pandemic, where it enabled early detection of outbreaks and complemented clinical surveillance systems [3,4,5]. The attraction of WBE derives from its numerous advantages, including its cost-effectiveness, independence from testing strategy and individual testing behaviour, and near real-time insights into the spread of infectious diseases, antimicrobial resistance (AMR), pharmaceutical use, and substance abuse patterns [6,7]. In addition, data collection is anonymous, and personal data cannot be misused for other purposes. The rapid identification of emerging threats through WBE supports robust data-driven public health interventions [8,9].

Despite the growing recognition of WBE as a valuable tool for public health surveillance, its adoption has faced challenges limiting its maximum performance. One is the lack of standardisation in sampling, sample processing, analytical methods, and data interpretation, which leads to inconsistencies across studies and jurisdictions [10,11,12]. Additionally, the sensitivity and precision of pathogen detection in wastewater are highly variable, influenced by factors such as sample matrix complexity, shedding rate, wastewater infrastructure, environmental conditions, and methodological differences [13]. These complicate data comparability over time and across regions, which reduces the reliability of WBE as a decision-making tool [14]. To address these gaps, initiatives such as the Global Consortium for Wastewater and Environmental Surveillance for Public Health (GLOWACON) have been launched. More specifically, GLOWACON aims to establish an international WBE sentinel system for harmonised early detection, prevention, and real-time monitoring of epidemic threats and outbreaks [15,16,17].

Finally, there is still a notable absence of centralised, curated databases that consolidate pathogen-specific information, metadata, and contextual variables necessary for robust epidemiological analysis [17,18]. In response to this absence, the *Encyclopaedia Cloacae* was developed.

The *Encyclopaedia Cloacae* represents an innovative and centralised resource for WBE. It seeks to enhance our understanding of transversal health matters and, consequently, our ability to respond to them. The advent of such a platform is timely, as it enables the early detection of threats—including novel pathogens—by systematically capturing and curating evidence from wastewater surveillance. This supports real-time monitoring and informs public health decision-making, thereby playing an integral role in pandemic preparedness [15,19]. The comprehensive database of pathogens and health hazards detectable in wastewater facilitates the collation and dissemination of critical data pertinent to researchers, public health officials, and policymakers [17]. Lastly, the *Encyclopaedia Cloacae* stands as a testimony to the collective efforts of the scientific community, which strives to maintain the accuracy and comprehensiveness of the database through structured and consistent data contributions.

This article provides a description of the *Encyclopaedia Cloacae* and outlines the workflow behind its development, maintenance, and updating, including the criteria used to guide the inclusion of pathogens. In addition, it showcases the complementary functionalities within the platform, such as the *Visualising the Invisible* dashboard. It discusses the added value and implications of the tool in a One Health context, an approach which recognises the interconnectedness of humans, animals, and the environment, and, finally, unveils directions for the future of the platform.

## 2. Materials and Methods

### 2.1. General Website Architecture

The *Encyclopaedia Cloacae* is hosted on the EU Wastewater Observatory for Public Health (EU4S) website and is continuously updated with the latest published literature. To achieve this, the platform has been meticulously developed and maintained through an administration (ADMIN) panel. It allows developers to easily populate the database and enhance its functionalities.

Data management is based on a robust data integration system, designed and implemented internally, employing a free and open-source Python-based web framework called Django (version 4.2) [20]. This tool has been integrated to work with the Zotero reference manager (https://www.zotero.org/, accessed on 26 May 2025), making it easy to add research papers and other publications to the main database [21]. The generated data is then handled with PostgreSQL (version 13.20), a powerful open-source object-relational database management system.

**Figure 1:**
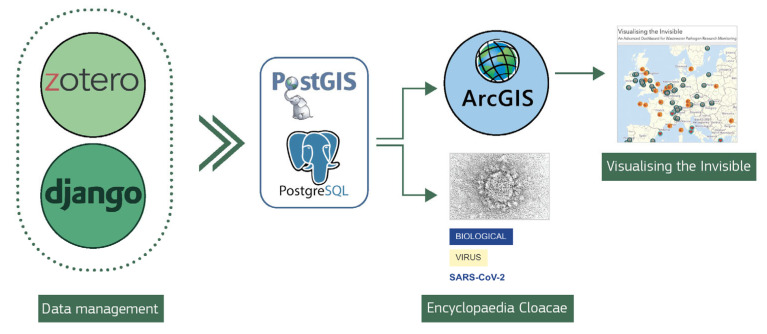
A scheme of the *Encyclopaedia Cloacae*’s architecture, illustrating the interactions between the components.

The *Visualising the Invisible* dashboard is an extension of the *Encyclopaedia Cloacae* and provides a visualisation of the most active WBE research locations. The ESRI ArcGIS Server dashboards (version 11.4) [22] and a PostGIS database (version 3.5.2) were used for its generation. The process of geo-referencing has been simplified through the use of gazetteers, databases that contain geographic information about places, cities, and administrative divisions. A detailed scheme of the architecture can be found in Figure 1, which illustrates the interactions between the different components.

Altogether, this architectural foundation enables semi-automatic connections between the synchronised publications and relevant entries within the *Encyclopaedia Cloacae*, thereby enhancing data linkages and fostering a more comprehensive understanding of the subject matter. All references integrated into the *Encyclopaedia Cloacae* are openly accessible under the terms and conditions of the Creative Commons Attribution (CC BY) license.

### 2.2. Criteria Description

Currently, the *Encyclopaedia Cloacae* operates using a set of three well-defined criteria that guide the inclusion and analysis of pathogens within the context of wastewater surveillance:

- Criterion 1: Detection in human excreta focuses on the detection of pathogens in various forms of human excreta, providing insights into transmission dynamics and potential disease spread.
- Criterion 2: Detection in wastewater emphasises the importance of detecting pathogens in wastewater and establishing reliable detection methods, crucial for effective surveillance and public health interventions.
- Criterion 3: Public health surveillance highlights the integration of pathogen detection into public health surveillance systems, supporting early detection and continuous monitoring of public health threats.

**Figure 2:**
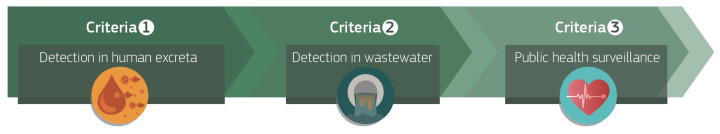
The criteria of the *Encyclopaedia Cloacae*.

The criteria were constructed ad hoc for the *Encyclopaedia Cloacae* by consulting peer-reviewed research [23,24,25,26] and later refined and validated through internal discussion with *Encyclopaedia Cloacae* partners and contributors. They serve as a framework for assessing the viability of specific pathogens to be monitored through wastewater analysis as well as for supporting public health interventions and decision-making. Together, they ensure that the information within the database is not only reliable, but also actionable, providing a significant advantage in the surveillance and containment of infectious diseases. The criteria, as represented in the *Encyclopaedia Cloacae*, are depicted in Figure 2.

#### 2.2.1. Criterion 1: Detection in Human Excreta

Criterion 1 focuses on the detection of pathogens in various forms of human excreta. This criterion is essential for understanding how pathogens are shed by infected individuals and subsequently enter wastewater systems. In addition, the importance of this criterion lies in its ability to provide a comprehensive picture of the pathways through which infections can spread within communities. For instance, pathogens present in faeces may indicate gastrointestinal infections, while those in respiratory secretions can point to respiratory disease. By means of this criterion, researchers can gain valuable insights into the transmission dynamics of diseases and the potential sources of outbreaks.

Furthermore, the data encompassed in this criterion supports the development of targeted public health interventions. By knowing which pathogens are prevalent in specific types of excreta, health officials can implement more effective monitoring and control measures. This can lead to timely responses to emerging health threats and better protection of the public.

The *Encyclopaedia Cloacae* quantifies the presence of pathogens in human excreta across eight harmonised categories, including faeces, urine, vomit, respiratory secretions, blood, genital secretions, tissues, and other body fluids (Table 1). The categories have been standardised for consistency. The list is not exhaustive and is continuously updated as new data on the detection of pathogens in human excreta becomes available in the literature.

#### 2.2.2. Criterion 2: Detection in Wastewater

Criterion 2 evaluates whether a pathogen is present in wastewater and whether reliable detection methods have been established through peer-reviewed research. Wastewater surveillance has been recognised as a rapid, sensitive, and cost-effective tool for monitoring various pathogens at the population level. Pathogens are introduced into the sewerage system through human excreta, including faeces, urine, sputum, and skin, as addressed in criterion 1. In view of this, criterion 2 proves crucial for understanding the potential for environmental detection of pathogens and the surveillance measures to be undertaken.

Furthermore, the presence of pathogens in wastewater serves as a key indicator of their distribution within communities. This information can contribute towards risk assessments and public health interventions aimed at mitigating the spread of diseases. Monitoring pathogens in wastewater can provide early warnings of disease outbreaks and help track the spatial and temporal trends of infections. As a result, health officials can identify emerging threats, allocate resources effectively, and implement timely public health interventions.

Nonetheless, for effective pathogen recognition in wastewater, reliable and validated detection methods, including microbiological and molecular techniques such as culture-based methods, quantitative PCR (qPCR), and next-generation sequencing (NGS), are indispensable. Considering this, detection methods should be continuously developed, improved, and adapted to fit their wide spectrum of applications.

#### 2.2.3. Criterion 3: Public Health Surveillance

Criterion 3 evaluates the integration of pathogen detection in wastewater into public health surveillance systems through pilot projects and research initiatives, or its incorporation into ongoing surveillance programs.

Despite the significant role of wastewater surveillance in achieving polio eradication and, more recently, in monitoring the COVID-19 pandemic, many global public health surveillance systems rely mostly on clinical data. Unlike wastewater-based approaches, these fail to provide a comprehensive picture of infection burden and community transmission. One pooled wastewater sample provides insights into the entire population of a catchment area, accounting for both symptomatic and asymptomatic individuals. In this regard, pathogens typically known to cause a high proportion of mild infections, those associated with nonspecific symptoms, and those leading to widespread poor health outcomes should be prioritised for wastewater monitoring programs. In addition, analysis of wastewater is valuable for diseases for which current diagnostic tests are inadequate, have limited accessibility, or are expensive, e.g., neglected tropical diseases.

Hence, the third criterion of the *Encyclopaedia Cloacae* provides valuable information on the effectiveness of wastewater as a tool for the early detection and continuous monitoring of public health threats, ultimately contributing to more effective public health interventions and improved population health outcomes. This criterion leverages the previous two criteria, making the information in the *Encyclopaedia Cloacae* not only descriptive, but also applicable.

### 2.3. Workflow: Population of Dataset and Maintenance

**Figure 3:**
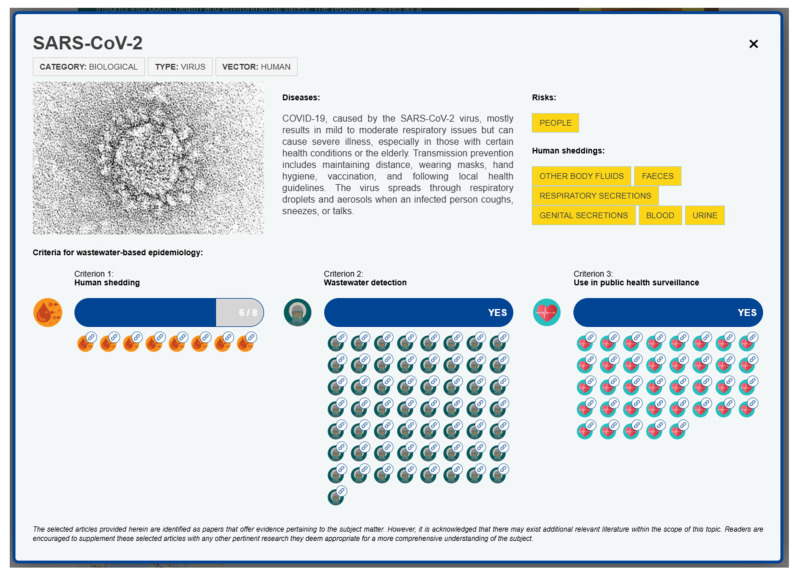
A screen clipping of the *Encyclopaedia Cloacae* depicting one of the pathogen factsheets.

The *Encyclopaedia Cloacae* relies on a systematic process to ensure its high quality, accuracy, and comprehensiveness. Procedures were designed to ensure consistent and standardised population of the database. Developers can add new pathogens, related peer-reviewed articles, and other accompanying information through the EU4S ADMIN panel. This facilitates the continuous updating and enhancement of the tool’s functionalities. Any new addition is subject to review and validation before being made publicly available. Furthermore, it is acknowledged that the set of research under each criterion is not exhaustive, and experts are welcome to contribute with their knowledge, peer-reviewed publications, and relevant pathogen images. Figure 3 shows a screen clipping of an *Encyclopaedia Cloacae* pathogen factsheet.

#### 2.3.1. Addition of Pathogens and Related Information

To add a new pathogen, developers must have specific information at hand to complete the operation:

- The name of the pathogen to be added.
- The type of pathogen (such as bacterium, virus, parasite, fungus, etc.).
- The vector, defined as the source of pathogen diffusion (such as animal, animal/human, human, environment, or none).
- A brief and easy-to-understand description (up to 500 characters), avoiding the use of acronyms.
- An optional thumbnail image with copyright clearance. If no image is available, a temporary generic clipart may be used.
- Information on the pathogen’s level of risk to humans, ecosystems, and/or farmed animals.

#### 2.3.2. Publication Entry

**Figure 4:**
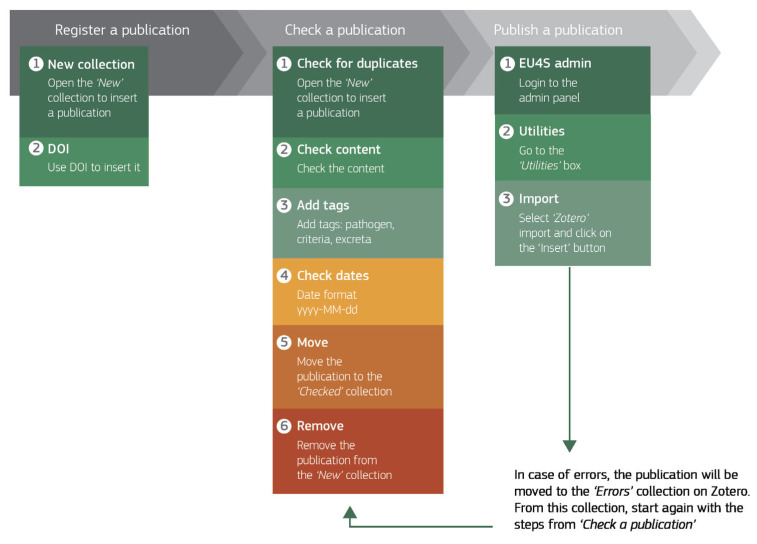
A summary scheme of the publication entry process. In Zotero, publications are added to a collection called ‘New’ using their DOI. The publications are checked for duplication, content, and date formatting. After tags are added specifying the pathogen, the criterion under which the publication falls, and, if necessary, the excreta in which the pathogen is shed, they are moved to the ‘Checked’ collection. From the EU4S ADMIN panel the publications are inserted into the website. In case of errors, the publications move to the ‘Errors’ collection for rechecking.

The standardised process for including peer-reviewed articles in the *Encyclopaedia Cloacae* database consists of four steps, namely, setting up the Zotero software (for first-time users), the registration of new items in Zotero, checking publication content, and publishing the references from the ADMIN panel. The workflow is outlined in Figure 4.

Articles are stored and organised in a specific ad hoc Zotero group library composed of six collections: New, Checked, Published, Errors, Info, and For Later. Their descriptions are provided in Table 2. Publications are ideally added to the “New” collection using a DOI or ISBN. These are preferred as Zotero currently uses these fields to determine duplicates.

Subsequently, newly added publications undergo content verification to determine whether they meet the *Encyclopaedia Cloacae* criteria for a selected pathogen. If the publication is deemed relevant, specific tags are assigned to enable Zotero to communicate with the EU4S ADMIN panel. The following three tags must be entered: pathogen, criteria, excreta. These are defined in Table 3. In case of missing categories, new pathogens or excreta can be added as needed directly through the ADMIN page.

After filling out the tag information, the publication is moved to the “Checked” collection, which automatically updates the visualisation of the publication on the EU4S ADMIN panel. Here, the publication entry process is finalised by publishing the pending publication. If no errors are reported upon publishing, this indicates that the publication has been successfully integrated into the *Encyclopaedia Cloacae*. In Zotero, published items are automatically moved from the “Checked” collection to the “Published” collection.

If an error is found, a warning message will appear indicating the type(s) of error. In this case, the publication will be moved to the “Errors” collection in Zotero. From here, users can re-add the publication to the “Checked” collection and correct the mistakes.

#### 2.3.3. Geo-Referencing Publications

Once the publications are made visible in *Encyclopaedia Cloacae*, geographic coordinates are assigned to the scientific studies. This information will go towards enriching the *Visualising the Invisible* dashboard, a visualisation of the most active WBE research hotspots. It is based on geolocating the added scientific references on the world map by study setting and/or author affiliation. The result is a visualisation with two possible modalities, one focused on highlighting the locations where research is being performed, the other focused on the location of the institutions fostering WBE research. Additional filters allow navigation according to criteria and pathogen type.

**Figure 5:**
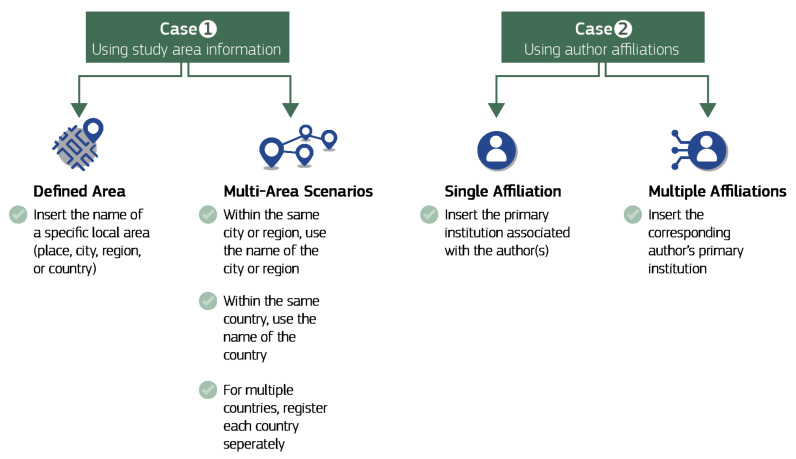
A summary scheme for geo-referencing publications.

The addition of geographic information sees two case scenarios, namely, the use of study area information and/or the use of author affiliation. It is recommended to add both when available. In the first case, the name of the specific local area (place, city, region, or country) is inserted as mentioned in the publication. In multi-area scenarios, the smallest common city, region, or country is registered. However, if the study spans multiple countries, each country is registered separately. As the study setting may not always be available, the affiliations of the authors are then to be used. In case of a single affiliation, the primary institution associated with the author(s) is entered, while multiple affiliations are handled by registering the primary institution of the corresponding author. Figure 5 provides a summary scheme of the two geo-referencing scenarios.

## 3. Results

**Figure 6:**
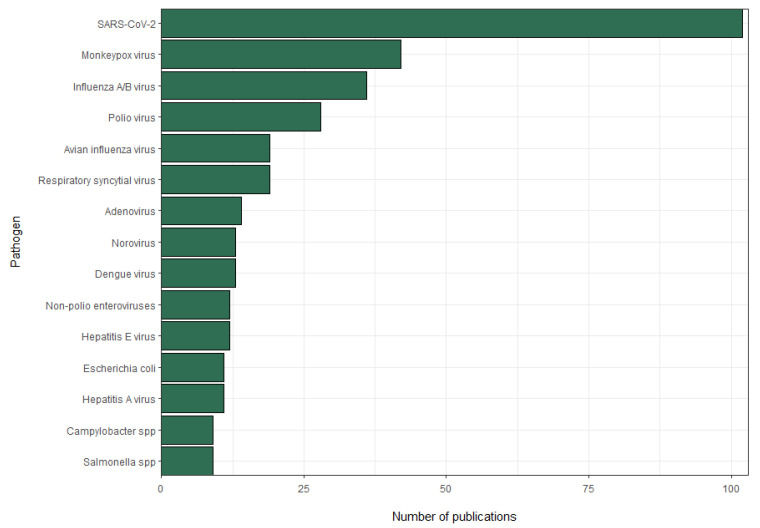
The ranking of the top 15 pathogens by number of publication entries.

At the time of writing, the *Encyclopaedia Cloacae* included 107 pathogens that spanned four microbial classes, namely viruses, bacteria, parasites, and fungi. The majority of pathogens considered were viruses (55/107; 51%), followed by bacteria and parasites (34/107 and 17/107; 32% and 16%, respectively). Fungi were represented by only one species, *Candidozyma auris* (formerly *Candida auris*). The 6 most referenced human pathogens were severe acute respiratory syndrome coronavirus 2 (SARS-CoV-2) (18%), influenza viruses (10%), Mpox virus (7%), polio virus (5%), respiratory syncytial virus (RSV) (3%), and adenovirus (3%). Other pathogens represented less than 3% of the total papers. For 4 pathogens, namely henipavirus, foot-and-mouth disease virus, *Echinococcus* spp., and *Trichinella spiralis*, no publications had been uploaded yet. Figure 6 shows the 15 pathogens with the most publication entries in the *Encyclopaedia Cloacae*. All raw data is available in Table S1.

Furthermore, the repository category contained a total of 331 publications, to support the three criteria under each pathogen. The raw count of entries was 564, including references that were added more than once as they met more than one criterion. Of these, 443 (77%) articles were specific to viruses, while only 93 and 36 (16% and 6%) considered bacteria and parasites, respectively. Only 2 papers documented fungal detection in wastewater, making up less than 1% of the total references.

**Figure 7:**
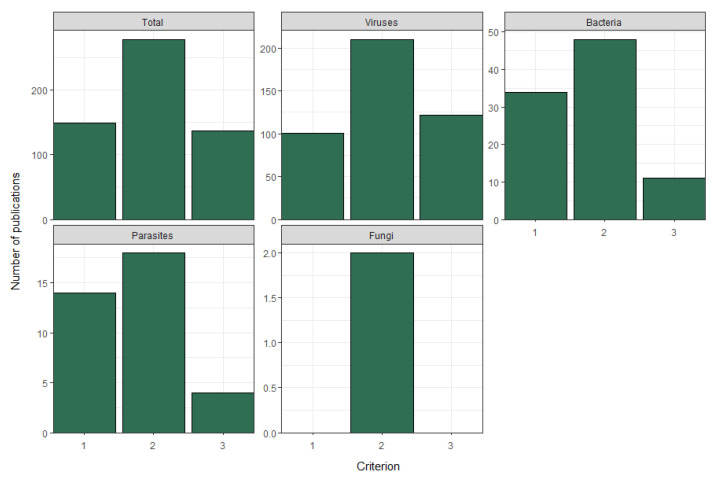
The number of publication entries per criterion, filtered by microbial class. Criterion 1 = detection in human excreta, criterion 2 = detection in wastewater, criterion 3 = public health surveillance.

When looking at the publication entries by criteria, most provided evidence for criterion 2, detection in wastewater (278/564; 49%). Criterion 1, detection in human excreta, and criterion 3, public health surveillance, were covered by almost the same number of publications, 149 and 137 (26% and 24%), respectively. The data is summarised in Table 4. When breaking down the coverage of each criterion by microbial class, all presented the same pattern reported previously, with viruses having the most extensive documentation, followed by bacteria, parasites, and fungi. It is of note that some publications met more than one criterion; hence, entry counts may contain duplicates. Findings are visualised in Figure 7.

Four vectors for the transmission of pathogens were identified, namely humans, animals, both, and the environment. Out of the 107 pathogens, 49 (46%) were reported to exclusively transmit between humans and 11 (10%) were transmitted only by animals. For 38 pathogens there was documentation for both human-to-human and animal-to-human transmission. Insects, such as mosquitoes, were considered as animals. Only 9 (8%) pathogens solely spread through the environment, e.g., *Legionella* spp. The data is shown in Table 5.

## 4. Discussion

In an era where infectious diseases transcend borders with unprecedented speed, the need for robust, population-level health surveillance tools is more urgent than ever [15,17]. The *Encyclopaedia Cloacae* addresses this need by offering not only a database of wastewater-detectable pathogens, but also a platform that bridges scientific knowledge with actionable public health strategies. It allows researchers, public health officials, policymakers, and other relevant actors to access and share critical insights on wastewater-detectable human hazards and, consequently, enhance their ability to react to them.

Although wastewater surveillance has been recognised as a powerful, cost-effective, and non-invasive lens to monitor community health in real time, many global public health surveillance systems still rely heavily on medically attended case data [24,27]. Unlike individual testing, wastewater testing offers insights into the entire population within a catchment area [24,27]. Therefore, infectious diseases that go under-reported by traditional clinical surveillance and have nonspecific symptoms, such as polio and influenza, and those for which current diagnostic tests are unavailable, inadequate, or expensive are ideal candidates for wastewater monitoring programs.

In this light, the *Encyclopaedia Cloacae* presents information on over 100 pathogens spanning the four most important microbial classes, namely viruses, bacteria, parasites, and fungi. Viruses were addressed by most publications, which is a limitation of the current dataset. This bias was most likely caused by the recent COVID-19 pandemic and the subsequent application of wastewater surveillance to other similar respiratory viruses. In fact, SARS-CoV-2 emerged as the best described pathogen within the *Encyclopaedia Cloacae*. In addition, the historic use of wastewater to monitor clusters of poliovirus infections and the recent global outbreaks of influenza, monkeypox, and RSV increased their documentation.

The *Encyclopaedia Cloacae* does not stop at being a mere bank of knowledge. What sets it apart is the triad of criteria constructed specifically for the *Encyclopaedia Cloacae*. These ensure that the information within the database is relevant and scientifically robust, but also actionable within public health frameworks. Criterion 1 focuses on the detection of pathogens in human excreta, criterion 2 on pathogen detection in wastewater, and criterion 3 on the integration of pathogen detection into public health surveillance systems. As of now, most publications within the database bring evidence to criterion 2, detection in wastewater. This highlights the current focus of WBE research, which primarily emphasises environmental detection. Criterion 1 is often restricted by the lack of consistently harmonised clinical shedding data, while criterion 3 is limited due to the relatively small number of studies that document the formal integration of WBE into surveillance frameworks. However, as the criteria were constructed to complement each other, all three need be populated equally. To fill these gaps, integrating *Encyclopaedia Cloacae*-related communications and EU4S monthly newsletters with the criteria could help clear up the type of contributions sought for the repository. In parallel, broader research efforts should encourage interdisciplinary collaboration, particularly by enhancing connections between environmental scientists and clinical researchers.

A more comprehensive triad of knowledge feeds into more effective pandemic preparedness, early warning of disease outbreaks, timely preventive measures, and strategic resource allocation. Relevant actors can draw from this knowledge to conceive new ideas, advancements, and collaborations. For example, authorities and politicians readily rely on national or international technology standards, as their structured approach and method validation ensure a strong consensus within a group of experts or stakeholders. The downside is that standardisation processes are comparatively slow, while usually the requirements for wastewater monitoring have to be implemented very quickly and dynamically [11,28]. Hence, the *Encyclopaedia Cloacae* can also offer the opportunity to obtain a relatively quick overview of the consensus among the current technologies used.

Although the *Encyclopaedia Cloacae* is not yet formally integrated into public health decision-making systems, it has been introduced to several national and international stakeholders, including public health institutes and environmental agencies. The platform is currently being explored as a supporting tool within the EU4S, with the aim of aligning environmental surveillance data with broader public health strategies. While integration with clinical data streams is not currently planned, the platform can be developed to be interoperable with other surveillance tools in the future, should the need arise. The ongoing cultivation of partnerships and the flexibility of the *Encyclopaedia Cloacae* will serve towards strategic decision-making and better protection of the public for improved health outcomes [15].

The systematic workflow on which the *Encyclopaedia Cloacae* relies ensures its high quality, accuracy, and comprehensiveness. The procedures are designed to guarantee the consistent and standardised population of the database. By making the workflow publicly available, authors promote transparency regarding how the published literature is utilised and processed as part of the website. Correct referencing and acknowledging the work of authors puts them at the centre of the knowledge base of the *Encyclopaedia Cloacae* and showcases their contribution to the scientific community.

With this, the *Encyclopaedia Cloacae* also seeks to introduce co-creation, advance co-ownership, and promote inclusion. The website’s facade was also designed to be intuitive for those less familiar with the field. To support this target, other functionalities such as the *Visualising the Invisible* dashboard were embedded into the platform to illustrate the extensive data in a single visualisation. In fact, the dashboard reflects the global distribution of knowledge on the topic and can quickly indicate where the most active research areas are situated but also where efforts are still lacking. In the future, overlaying this existing visualisation with a problem map, which identifies populations and settings of concern with regard to health threats, would be highly useful. Knowing the critical locations requiring intervention and the research hubs with know-how can help direct resources where they are most needed. In the long run, this approach can drive more equitable research practices while stimulating the improvement of public health globally [29].

Currently, the *Visualising the Invisible* dashboard cannot be used to draw such conclusions as the database reflects only a subset of the available literature. In addition, many gaps in global research persist, especially in low-resource settings where wastewater surveillance could have the most impact. While the *Encyclopaedia Cloacae* stands as a testimony to the collective efforts of the scientific community to create a complete wastewater-detectable pathogen repository, stronger partnerships and collaborations are needed moving forwards in order to achieve this to the fullest. This union of forces sees no national boundaries, embraces under-resourced regions, and exercises a multidisciplinary approach. That being said, readers and website visitors are highly encouraged to supplement the *Encyclopaedia Cloacae* with any other pertinent research for a more comprehensive understanding of the subject.

In terms of future developments of the *Encyclopaedia Cloacae*, short-term efforts are going towards the enhancement of the existing pathogen database in terms of geographical and microbial class coverage. In addition, it is planned to introduce other facades to the *Encyclopaedia Cloacae*, namely AMR-related entries in the medium term and human biomarkers, chemical compounds, toxins, and other health determinants relevant to One Health surveillance in the long term. Finally, the future of the *Encyclopaedia Cloacae* strives for the full automation of its publication entry and geo-referencing workflow. The integration of artificial intelligence, machine learning, and deep learning tools can revolutionise the platform, enabling a dynamic, real-time snapshot of the global wastewater landscape.

With these developments, the ultimate ambition of the *Encyclopaedia Cloacae* is to be a mirror to the health of society. Health goes beyond humans and spans across a range of disciplines and sectors [17]. In fact, it also embraces animals and the whole ecosystem, as defined by the One Health approach, as well as aspects of health and environmental safety. As people are increasingly on the move, health threats quickly become transnational hazards. Each entry in the encyclopaedia tells a bit more about hazards, their detection, and their impact on our world, but, more importantly, each entry serves as a call to action to invest in wastewater-based health surveillance, embrace collaborative knowledge-sharing, and take prompt action before the advent of irreversible disease scenarios. Its evolution will be critical in shaping resilient health systems capable of meeting current and future public health challenges.

## Figures and Tables

**Table 1:** A list of the proposed harmonised categories for human shedding.

Proposed Harmonised Categories	Existing Categories
Faeces	Rectal bleeding
	Faeces
	Stool
	Rectal swabs
Urine	Urine
Vomit	Vomit
Respiratory secretions	Mucus
	Nasal throat
	Nasal secretions
	Respiratory fluids
	Respiratory
	Respiratory droplets
	Respiratory specimens
	Saliva
	Throat secretions
	Sputum
	Spittle
Blood	Blood
	Serum
	Plasma
Genital secretions	Semen
	Vaginal fluids
Tissues	Tissues
	Skin
Other body fluids	Cerebrospinal fluid
	Sweat
	Breast milk
	Tears
	Fluids or excretions from abscesses

**Table 2:** Descriptions of the Zotero folders.

Name	Description
New	New publications not yet reviewed.
Checked	Publications reviewed for content and linked to hazard(s) and criteria.
Published	Folder automatically populated with publications registered on the website.
Errors	Publications rejected from automatic registration on the website.
Info	Contains reference materials such as the guidance manual and lists of pathogens and excreta.
For Later	Stores publications for future developments of the *Encyclopaedia Cloacae*.

**Table 3:** Descriptions of the Zotero tags.

Name	Description
pathogen	The name of the pathogen(s), separated by commas and registered according to standard nomenclature.
criteria	The *Encyclopaedia Cloacae* criterion or criteria the publication refers to, separated by commas.
excreta	Required only for criterion 1. The excreta where the selected pathogen has been found, separated by commas and registered according to the harmonised excreta categories reported in Table 1.

**Table 4:** The number of pathogens included per microbial class and the raw count of publications under each criterion, filtered by microbial class.

Microbial Class	Number of Pathogens (%)	Number of Publications (%)			
		Criterion 1: Detection in Human Excreta	Criterion 2: Detection in Wastewater	Criterion 3: Public Health Surveillance	Total
Viruses	55 (51)	101 (68)	210 (76)	122 (89)	433 (77)
Bacteria	34 (32)	34 (23)	48 (17)	11 (8)	93 (16)
Parasites	17 (16)	14 (9)	18 (6)	4 (3)	36 (6)
Fungi	1 (1)	0	2 (1)	0	2 (<1)
Total	107	149 (26)	278 (49)	137 (24)	564

**Table 5:** The number of pathogens per disease transmission vector, filtered by microbial class.

Microbial Class	Number of Publications (%)				
	Human	Animal/Human	Animal	Environment	Total
Viruses	36 (34)	13 (12)	4 (4)	2 (2)	55 (51)
Bacteria	13 (12)	14 (13)	4 (4)	3 (3)	34 (32)
Parasites	0	10 (9)	3 (3)	4 (4)	17 (16)
Fungi	0	1 (1)	0	0	1 (1)
Total	49 (46)	38 (36)	11 (10)	9 (8)	107

## Data Availability

The original data presented in the study are openly available in the *Encyclopaedia Cloacae* at https://wastewater-observatory.jrc.ec.europa.eu/#/encyclopaedia-cloacae (accessed on 26 May 2025) under the Creative Commons Attribution (CC BY) license (https://creativecommons.org/licenses/by/4.0/, accessed on 26 May 2025). A complementary *Visualising the Invisible* dashboard is also accessible at https://wastewater-observatory.jrc.ec.europa.eu/#/gis-area/8 (accessed on 26 May 2025).

## Supplementary Materials

The following supporting information can be downloaded at: https://www.mdpi.com/article/10.3390/microorganisms13081900/s1, Table S1: raw data.

## Abbreviations

The following abbreviations are used in this manuscript:

ADMIN	Administration
AI	Artificial intelligence
AMR	Antimicrobial resistance
DOI	Digital Object Identifier
EU4S	EU Wastewater Observatory for Public Health
ISBN	International Standard Book Number
NGS	Next-generation sequencing
qPCR	Quantitative polymerase chain reaction
RSV	Respiratory syncytial virus
SARS-CoV-2	Severe acute respiratory syndrome coronavirus 2
WBE	Wastewater-based epidemiology

## Appendix A

*Encyclopaedia Cloacae* collaborators:

- Giuseppina La Rosa—Department of Environment and Health, Istituto Superiore di Sanità, Rome, Italy.
- Elisabetta Suffredini—Department of Food Safety, Nutrition and Veterinary Public Health, Istituto Superiore di Sanità, Rome, Italy.
- Timo Greiner—Robert Koch Institute, Berlin, Germany.
- Sophia Beyer—Department of Infectious Diseases, Robert Koch Institute, Berlin, Germany.
- Timo Greiner—Department of Infectious Disease Epidemiology, Robert Koch Institute, Berlin, Germany.
- Sophie Schneitler—Institute of Pneumology, University of Cologne, Köln, Germany.
- Sören L. Becker—Institute of Medical Microbiology and Hygiene, Saarland University, Homburg, Germany.
- Dr. Hagen Reichert—Gemeinschaftspraxis für Kinder- und Jugendmedizin, Homburg, Germany.
- Ulrike Braun—Umweltbundesamt, Berlin, Germany.
- Bilge Alpaslan Kocamemi—Turkish Water Institute (SUEN); Faculty of Engineering, Environmental Engineering Department, Marmara University, Istanbul, Turkey.
- Kata Farkas—School of Environmental and Natural Sciences, Bangor University, Bangor, United Kingdom.
